# An all-out assault on SARS-CoV-2 replication

**DOI:** 10.1042/BCJ20210256

**Published:** 2021-07-02

**Authors:** Ronald T. Hay

**Affiliations:** Centre for Gene Regulation and Expression, School of Life Sciences, University of Dundee, Dow Street, Dundee DD1 5EH, U.K.

**Keywords:** antivirals, coronavirus, drugs, RNA replication

## Abstract

The coronavirus pandemic has had a huge impact on public health with over 165 million people infected, 3.4 million deaths and a hugely deleterious effect on most economies. While vaccination effectively protects against the disease it is likely that viruses will evolve that can replicate in hosts immunised with the present vaccines. Thus, there is a great unmet need for effective antivirals that can block the development of serious disease in infected patients. The seven papers published in this issue of the Biochemical Journal address this need by expressing and purifying components required for viral replication, developing biochemical assays for these components and using the assays to screen a library of pre-existing pharmaceuticals for drugs that inhibited the target *in vitro* and inhibited viral replication in cell culture. The candidate drugs obtained are potential antivirals that may protect against SARS-CoV-2 infection. While not all the antiviral candidates will make it through to the clinic, they will be useful tool compounds and can act as the starting point for further drug discovery programmes.

## Commentary

The public health and economic impact of the severe acute respiratory syndrome coronavirus type 2 (SARS-CoV-2) pandemic has been enormous. As of 21st May 2021, there were over 165 million confirmed cases, 3.4 million deaths and many millions left with long-term illness. Aside from public health measures to reduce transmission, 1.6 billion vaccine doses have been administered (statistics from The John Hopkins University Centre for Systems Science and Engineering) and in countries where vaccination rates are high, this appears to be stemming the tide of deaths and serious illness resulting from infection. However, it is clear that rapid mutation of the virus in areas where infection rates are high will lead to the emergence of viral variants that are no longer controlled by the existing vaccines. Until large scale production of vaccines that neutralise the new variants comes on stream there will be a gap in protection when there will be a requirement for antiviral compounds that ameliorate the consequences of infection. To date, the only antiviral used to treat patients infected with SARS-CoV-2 is the nucleoside analogue remdesivir that inhibits the viral RNA dependent RNA polymerase (RdRp). Originally developed to treat Ebola infection it has emergency use authorisation for the treatment of SARS-CoV-2 infection and is being widely tested in a clinical setting. The search for antiviral compounds is being conducted on a global scale in academic, biotech and big pharma laboratories and it is to this end that a co-ordinated effort led by the biochemist John Diffley of the Francis Crick Institute was established to search for existing drugs that would suppress replication of SARS-CoV-2. Teams were assembled from within the Francis Crick Institute, the University of Dundee and University College, London that would each be responsible for identifying a component of the SARS-CoV-2 replication machinery, purifying the protein and developing assays to assess the biochemical activity. Once this had been accomplished high-throughput assays were developed and used to screen a custom compound library of 5000 previously characterised pharmaceuticals. The advantage of repurposing drugs developed for other targets is the dramatically reduced time from testing to the clinic. Approved or investigational drugs will have accumulated considerable data on drug chemistry, pharmacology and toxicology in human patients. This would facilitate rapid passage through clinical trials and regulatory procedures. Once candidate compounds were identified in the screen they were then tested for their ability to inhibit viral replication in tissue culture using a plaque assay for infectivity. Compounds that inhibited viral replication without deleterious effects on uninfected cells were selected as candidate antivirals. The seven papers describing these results are published in this addition of the Biochemical Journal [1–7].

**Figure 1.**
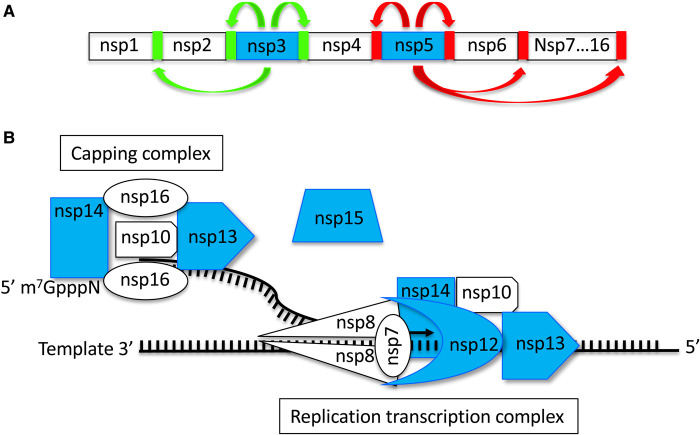
Coronavirus non-structural proteins (nsps). (**A**) The N-terminal region of the polyprotein showing the two viral proteases (blue) with green arrows indicating the sites of cleavage mediated by the nsp3 papain-like protease (PL^pro^) and sites of cleavage of the nsp5 chymotrypsin-like protease (also known as 3CLpro or Main protease Mpro) in red. (**B**) The capping complex and replication transcription complex with the associated uridine specific endoribonuclease nsp15. Components studied in the drug discovery studies [1–7] indicated in blue.

SARS-CoV-2 is an enveloped virus and contains a large (30 kb) positive-sense, single-stranded RNA genome. These viruses gain entry to their target cells by virtue of their Spike proteins that decorate the viral membrane and engage the angiotensin-converting enzyme 2 (ACE2) as a receptor to gain access to the cell. The large viral mRNA is capped at its 5′ end and polyA modified at its 3′ end and as it is positive sense can be directly translated by cellular ribosomes. Within the genome are 14 open reading frames (ORFs) that encode the non-structural proteins (nsp) in two large ORFs that cover the 5′ two-thirds of the genome and 12 smaller ORFs in the 3′ third of the genome that encode structural and accessory proteins translated from sub-genomic RNAs. The two large ORFs, ORF1a and ORF1b are translated into polyprotein pp1a and polyprotein pp1ab that is a C-terminally extended form of pp1a generated as a consequence of a -1 ribosomal frameshift. These polyproteins are cleaved into 16 nsps by two viral proteases that excise themselves from pp1a (Figure 1a). Nsp5 is a chymotrypsin-like protease (also known as 3CL^pro^ or Main protease M^pro^) while the papain-like protease (PL^pro^) is located in nsp3. The remaining 14 nsps carry out viral genome replication, transcription and mRNA modification in addition to manipulating the host cell environment to facilitate viral replication. The first stage in genome replication is the generation of the complementary negative-sense RNA or anti-genome that can serve as a template for the synthesis of new genomes and for the transcription of viral mRNAs. Both of these functions are carried out by the viral RdRp or nsp12, the catalytic core of the replication–transcription complex (RTC) (Figure 1b). This complex is embedded in membranous structures that are hijacked from the endoplasmic reticulum and serve as factories for viral replication. Based on homologous proteins in other coronaviruses, it is thought that the SARS-CoV-2 RTC is anchored on the membrane by transmembrane domains located in nsps 3, 4 and 6. Many of the nsps, the nucleocapsid protein and many host proteins are recruited into the RTC. Key activities of the RTC are the synthesis and capping of the viral RNAs. Capping is mediated by a subcomplex of nsps 10, 13, 14 and 16 while RNA synthesis is mediated by nsps 7, 8, 10, 12, 13 and 14 (Figure 1b). At the core of the synthesis, the complex is the RdRp (nsp12), its processivity factors nsp7 and 8 and the RNA helicase activity of nsp13. With such a large genome the virus requires a proofreading exonuclease (nsp14) to remove mispaired bases incorporated by the error-prone RdRp. Capping of viral RNA requires the concerted action of nsp13, an unknown GTPase, the N7 methyltransferase nsp14 and the 2′-O-methyltransferase nsp16 with its nsp10 cofactor [8,9]. Viral RNA is capped not only to ensure efficient translation but also to stop pattern recognition receptors (PRRs) from seeing uncapped mRNA as a pathogen associated molecular pattern (PAMP) and triggering an innate immune response that would block viral replication. Likewise, it is thought that the uridine specific nsp15 endoribonuclease associates with the RTC and removes polyU from the 5′end of negative-strand RNA, which would otherwise trigger an interferon response.

The strategy take by the team was to target the two viral proteases required for excision of the nsps from the initially translated polyproteins, the RNA synthesis machinery, components of the capping complex and the nsp15 endoribonuclease (Figure 1b). All these proteins are essential genes, are mutationally constrained and can be assayed biochemically. They thus represent excellent targets for antiviral drugs. The product of nsp14 is a bifunctional enzyme with an N-terminal 3′-5′ exoribonuclease (ExoN) domain and a C-terminal S-adenosylmethionine-dependent N7 methyltransferase domain. As nsp14 is associated with both the replication transcription and the capping complexes this is consistent with the involvement of the ExoN activity being involved in proofreading during replication/transcription and the N7 methyltransferase activity being involved in capping. While it can be advantageous for viruses to accumulate mutations to escape from immune surveillance and become resistant to drugs, the accumulation of many mutations in a large genome could lead to loss of fitness. Drugs against the proofreading activity could be very useful as it is this very activity that can remove nucleotide analogue chain terminators after they have been incorporated into the growing RNA chain, thus reducing the effectiveness of these antiviral drugs. It was demonstrated [1] that Patulin, a lactone mycotoxin that had previously been tested against the common cold, inhibited the ExoN activity of nsp14 and reduced viral replication, although some toxicity was observed. Aurintricarboxylic acid, a known nuclease inhibitor, appeared to have specificity for Nsp14 and also inhibited viral replication. The other activity of Nsp14 is a guanine N7 methyltransferase involved in the capping of viral RNAs. It was shown [2] that many known drugs inhibited this activity: Lomeguatrib, an inhibitor of O6-methyl guanine DNA methyltransferase that enhances the effect of DNA alkylating agents in cancer treatment; Trifluperidol, used in the treatment of psychoses; IF-03882845 a non-steroidal mineralocorticoid antagonist used to treat diabetic nephropathy. While all inhibited viral replication and had synergistic interactions with remdesivir the severe side effects associated with Trifluperidol may limit its use as a single agent. A series of compounds related to suramin were shown to inhibit the RdRp encoded by nsp12 in complex with its processivity factors nsps 7 and 8 [3]. Suramin was developed over 100 years ago by Bayer for the treatment of river blindness and sleeping sickness and is known to inhibit multiple viruses. GSK-650394 also inhibited the RdRp and blocked SARS-CoV-2 replication. Its intended target is serum and glucocorticoid-activated kinase (SGK1 and 2) but it also inhibits influenza virus replication. Involved in both replication/transcription and capping the Nsp13 RNA helicase was shown to be inhibited by FPA-124, a cell-permeable Akt inhibitor and like the RdRp was also inhibited by several suramin related compounds [4]. Although both inhibited viral replication in cell culture experiments it remains to be established if inhibition is via Nsp13. The Nsp15 uridine specific endoribonuclease was shown to be inhibited by NSC95397 *in vitro* but it did not appear to exert any inhibitory effect *in vivo* [5]. Protease inhibitors are effective antivirals and have been licenced for the treatment of human immunodeficiency virus (HIV) and hepatitis C virus [10]. The two SARS-CoV-2 proteases (Figure 1a) were thus attractive targets for the development of antiviral drugs. The main protease Nsp5 was inhibited by Calpain inhibitor 1 which also reduced viral infectivity in cell culture. Peptidyl fluoromethylketones that mimicked the peptide cleavage site in the virus polyprotein were highly specific and potent inhibitors *in vitro* but less so *in vivo* [6]. They represent excellent starting points for further drug development. PL^pro^ the papain-like protease encoded in Nsp3 was inhibited by Dihydrotanshinone a natural compound from *Salvia miltiorrhiza* used in Chinese medicine. It inhibits the cleavage of viral peptides and inhibits isopeptidase activity against ubiquitin and ISG15. In cell culture, it blocks viral replication with no observable toxicity [7].

It is important to have multiple drug candidates. One reason for this is the attritional nature of any drug discovery programme. Many drugs that are effective in a tissue culture setting will fall by the wayside during animal testing and clinical trials. The other reason is that drugs against multiple targets may be necessary to counteract the development of drug-resistant mutants. The simultaneous delivery of multiple drugs is the strategy that has been used successfully to treat acquired immune deficiency syndrome (AIDS) caused by HIV. Given the rate at which mutations accumulate in the HIV genome, the administration of a single drug rapidly results in the development of resistance. However, the administration of a cocktail of three different drugs hitting different targets makes it very difficult for the virus to simultaneously acquire resistance to all three drugs. As SARS-CoV-2 replication/transcription is associated with a proofreading activity and causes an acute infection compared with the persistent HIV infection and it may be that SARS-CoV-2 would accumulate less mutations than HIV. However, it would certainly be desirable to have an arsenal of drugs inhibiting different targets to provide clinicians with a range of options when treating SARS-CoV-2 infected patients.

The next stage for these drug candidates is that they will need to be tested to determine their ability to block SARS-CoV-2 infection and pathogenicity *in vivo*. Initially, this could be done in transgenic mice expressing human ACE2. Upon intranasal inoculation with SARS-CoV-2 these mice develop a pulmonary infection that appears to mimic the pathological changes seen in patients infected with SARS-CoV-2 [11]. If the candidate drugs are successful in this setting they would need to be tested in non-human primates before moving on to clinical trials.

While many of the drug candidates with antiviral activity may not make it through to the clinic they can be very useful tool compounds for investigators studying SARS-CoV-2 replication. The ability to block the activity of non-structural components of the virus provides the researcher with the means to dissect the viral replication cycle. These drug candidates can also act as the starting point for further drug discovery programmes. Many the nsps targeted in the accompanying papers have already had their 3D structures determined by X-ray crystallography or cryoelectron microscopy [12–21]. Determination of the drug nsp complexes would allow the drug candidates to be optimised for binding specificity and affinity. This study also provides an excellent resource for other investigators working in this area to obtain expression constructs and assay details.

## Abbreviations

ACE2: angiotensin-converting enzyme 2
ExoN: exoribonuclease
HIV: human immunodeficiency virus
nsp: non-structural proteins
ORFs: open reading frames
RdRp: RNA dependent RNA polymerase
RTC: replication–transcription complex
SARS-CoV-2: severe acute respiratory syndrome coronavirus type 2
